# Seed-based resting-state connectivity as a neurosignature in fibromyalgia and depression: a narrative systematic review

**DOI:** 10.3389/fnhum.2025.1548617

**Published:** 2025-04-28

**Authors:** Betina Franceschini Tocchetto, Andrea Cristiane Janz Moreira, Álvaro de Oliveira Franco, Iraci L. S. Torres, Felipe Fregni, Wolnei Caumo

**Affiliations:** ^1^Post-Graduate Program in Medical Sciences, School of Medicine, Federal University of Rio Grande do Sul (UFRGS), Porto Alegre, RS, Brazil; ^2^Laboratory of Pain and Neuromodulation, Hospital de Clínicas de Porto Alegre (HCPA), Porto Alegre, RS, Brazil; ^3^Pain and Palliative Care Service, Hospital de Clínicas de Porto Alegre (HCPA), Porto Alegre, RS, Brazil; ^4^Service of Neurology, Hospital de Clinicas de Porto Alegre, Universidade Federal do Rio Grande do Sul, Porto Alegre, Brazil; ^5^Post-Graduate Program in Biological Sciences: Biochemistry, Universidade Federal do Rio Grande do Sul, Porto Alegre, Brazil; ^6^Laboratory of Pharmacology in Pain and Neuromodulation: Pre-clinical Investigations, Experimental Research Center, HCPA, Porto Alegre, Brazil; ^7^Laboratory of Neuromodulation and Center for Clinical Research Learning, Physics and Rehabilitation Department, Spaulding Rehabilitation Hospital, Boston, MA, United States; ^8^Department of Surgery, School of Medicine, Federal University of Rio Grande Do Sul (UFRGS), Porto Alegre, Brazil

**Keywords:** fibromyalgia, depression, functional connectivity, fMRI, neuroimaging fibromyalgia, neuroimaging

## Abstract

**Background:**

Major depressive disorder (MDD) often co-occur with fibromyalgia (FM), and both conditions have been associated with impaired resting state functional connectivity (rs-FC). The present systematic review aims to summarize the evidence on rs-FC in individuals with MDD and FM compared with healthy controls and explore overlapping connectivity patterns and their relationships with clinical symptoms.

**Methods:**

A systematic search of the EMBASE, PubMed, Scopus and ScienceDirect databases was conducted according to PRISMA guidelines. Studies were included that addressed rs-FC using seed-based analysis in MDD and FM patients compared to HC. Methodological quality and risk of bias were assessed using a 13-point checklist adapted from previous neuroimaging meta-analyzes.

**Results:**

A total of 33 articles were included in the analysis (17 with MDD and 16 with FM). The sample comprised 1,877 individuals, including 947 patients and 930 controls, with a mean age of 39.83 years. The seeds were categorized into six neural networks. Shared disruptions across MDD and FM studies have been identified in key circuits, including decreased connectivity between the insula and anterior cingulate cortex (ACC), middle frontal gyrus (MFG), superior frontal gyrus (SFG), and putamen. Increased FC was observed between the dorsolateral prefrontal cortex (DLPFC) and ACC, as well as between the thalamus and precuneus. Decreased insula-ACC connectivity correlated with greater pain intensity and catastrophizing in FM and with more severe depressive symptoms in MDD. Unique patterns of rs-FC were also observed: FM-specific changes involved the periaqueductal gray, hypothalamus, and thalamus, indicating impaired pain modulation and emotional processing. In contrast, MDD-specific changes were primarily observed in the reward, salience, and default mode networks, reflecting impaired emotional regulation. The studies showed considerable heterogeneity in the selection of seeds and study designs, which limits the feasibility of meta-analyses and underlines the need for standardized methods.

**Findings:**

This study provides information about overlapping and distinct neural mechanisms in FM and MDD, suggesting potentially the presence of a potential neurosignature that reflects shared disruptions in pain and emotion regulation networks while highlighting unique pathways underlying their respective pathophysiology.

## 1 Introduction

Fibromyalgia (FM), a chronic pain syndrome, is a global health concern with a prevalence estimated at 2–3% (Jones et al., 2015; Sarzi-Puttini et al., 2020). This condition, characterized by a wide range of symptoms, including widespread musculoskeletal pain, fatigue, cognitive-emotional disturbances, and non-restorative sleep (Wolfe et al., 2016; Siracusa et al., 2021), is often accompanied by high rates of psychiatric comorbidities, significantly contributing to patient distress (Hudson et al., 1992; Fietta et al., 2007; Kleykamp et al., 2021). One of the most prevalent comorbidities is Major Depressive Disorder (MDD), affecting more than half of FM patients, with a weighted prevalence of up to 63% (Kleykamp et al., 2021).

MDD is a debilitating psychiatric disorder influenced by a combination of biological, psychological, and socioeconomic factors (Kendler and Karkowski-Shuman, 1997; Cui et al., 2024). It is characterized by persistent low mood, reduced ability to derive pleasure from daily activities, cognitive impairments, and vegetative symptoms (Malhi and Mann, 2018). Alongside depression and anxiety, catastrophizing, an exaggerated negative response to pain, has been linked to increased brain activity in regions responsible for the anticipation, attention, and emotional processing of pain (Gracely et al., 2004). These findings heightened cortical response may affect the sensory processing of pain, as observed in patients with chronic pain (Yunus, 2008; Kaltsas and Tsiveriotis, 2023), and may lead to alterations in the functional connectivity (FC) of brain regions involved in the affective-motivational aspects of pain processing (Mouraux et al., 2011). Neuroimaging evidence has revealed structural and functional brain alterations across various neuronal circuits in MDD and FM patients (Flodin et al., 2014; Fallon et al., 2016; Runia et al., 2022; Sun et al., 2024). Resting-state functional magnetic resonance imaging (rs-fMRI), first introduced by Biswal et al. (1995), is used to assess functional connectivity (FC) between brain regions by examining statistical dependencies in neural activity (Fox and Raichle, 2007). Seed-based analysis, a commonly employed method, identifies FC abnormalities by examining the relationship between a seed region (a predefined area of interest) and other brain regions (Fox and Raichle, 2007). This method is particularly relevant in clinical research, as it enables the investigation of specific hypotheses by targeting the brain regions involved in pain processing and emotional regulation (Metwali and Samii, 2019). While it is limited by its sensitivity to seed selection (Bijsterbosch et al., 2017), this method remains crucial in investigating FC alterations in clinical populations, providing insights into the neural mechanisms underlying various disorders. This relationship is expressed in pathological conditions through clinical symptoms (Napadow et al., 2010; Wise et al., 2014). In MDD, FC alterations are often observed in frontal-subcortical pathways involving regions such as the dorsolateral prefrontal cortex (DLPFC), orbitofrontal cortex, anterior cingulate cortex (ACC), insula, hippocampus, amygdala, and thalamus (Ambrosi et al., 2017; Connolly et al., 2017; Zhao et al., 2022; Tu et al., 2024; Zhou et al., 2024). Resting-state (rs)-FC studies with FM patients consistently show alterations in cognitive and emotional networks, which are closely linked to the exacerbation of clinical symptoms, such as heightened pain perception, pain catastrophizing, and affective distress (Ichesco et al., 2014; Kong et al., 2019; Park et al., 2022). Greater connectivity between the insula and cingulate cortex has been associated with decreased pressure-pain thresholds, highlighting the neural basis of pain hypersensitivity in FM (Ichesco et al., 2014, 2016). Additionally, disruptions in the pain modulatory system, particularly in the descending pain inhibitory network, have been well-documented (Jensen et al., 2012). These include alterations in the periaqueductal gray (PAG), a critical hub for descending pain modulation *via* projections to the nucleus raphe magnus and spinal cord (Truini et al., 2016), and sensorimotor areas (Flodin et al., 2014; Pujol et al., 2014), which are primarily involved in the discriminative domain of pain through interactions with the thalamus and brainstem (Kim et al., 2021). Despite these insights, findings from studies comparing rs-FC in MDD and FM remain heterogeneous.

A meta-analysis of rs-FC in MDD found reduced connectivity in the frontoparietal network and increased connectivity in the default mode network (DMN) (Kaiser et al., 2015). In contrast, a study involving 1,300 participants observed reduced FC in the DMN only in individuals with recurrent MDD, suggesting that prolonged disease burden may contribute to these alterations and highlight the complexity of the neurocircuitry of depression (Yan et al., 2019). In patients with FM, altered FC between the insula and DMN has been linked to reduced clinical pain (Napadow et al., 2012). Moreover, studies of the cognitive control network in FM have found increased FC between the DLPFC and the ACC/medial prefrontal cortex (mPFC), which are regions involved in the top-down modulation of pain (Kong et al., 2019). Hyperconnectivity in these areas correlates with clinical symptoms, including pain catastrophizing (Galambos et al., 2019). In addition to the diverse findings regarding FC alterations, further research is required to integrate these results and provide a more comprehensive understanding of rs-FC disruptions across brain regions in patients with depression and FM. This systematic review aimed to investigate rs-FC alterations in individuals with MDD and FM using seed-based analysis compared to healthy controls (HC). Additionally, the review sought to identify common or overlapping FC patterns between the two conditions and examine their correlations with clinical symptoms. Through this analysis, we aimed to offer new insights that can inform future research and contribute to developing personalized therapeutic approaches.

## 2 Methods

This systematic review followed the Preferred Reporting Items for Systematic Reviews and Meta-analyses (PRISMA) guidelines (Page et al., 2021). The protocol details are registered in the PROSPERO International Prospective Register of Systematic Reviews (CRD42024573260).

### 2.1 Search strategy

We searched for relevant studies in PubMed, EMBASE, Scopus, and ScienceDirect databases through June 19, 2024. The MeSH or Entree terms used, and their combinations, were as follows: (“Fibromyalgia” OR “Depression”) AND (“Resting-state functional connectivity” OR “fMRI”) AND “Adults” AND “Observational Studies”.

To avoid bias, two independent researchers manually examined the references of the selected studies and those from previous reviews or meta-analyses. The specific search terms used for each electronic database are provided in Supplementary material.

### 2.2 Literature selection criteria

We included published original articles if they met the following criteria: (1) studies involving human subjects; (2) written in English, Spanish, or Portuguese; (3) observational studies or randomized clinical trials with baseline data and a healthy matched control group (HC); (4) focused on a patient group with a clear diagnosis of FM or MDD; (5) directly compared resting-state functional connectivity (rs-FC) using fMRI between the group, FM/MDD patients vs. HC; (6) utilized rs-FC seed-based analysis; and (7) studies that reported increases and decreases in rs-FC compared groups. No filter was applied concerning the publication year. Only between-group results at baseline were included in clinical randomized trials and cohort studies.

The exclusion criteria were as follows: (1) studies with other chronic pain or other psychiatric diagnoses; (2) patients with remitted or subthreshold depression; (3) the presence of MDD subgroups; (4) studies focusing exclusively on older adults (75+) due to alterations in connectivity associated with brain aging (Ferreira and Busatto, 2013) and the frequent association of depression with cognitive impairment in this population (Xie et al., 2013); and (5) studies involving children and adolescents (under 18 years) due to ongoing brain connectivity development in this age group (Sanders et al., 2023).

### 2.3 Selection process

Two authors independently and blindly screened and selected the studies (titles and abstracts) using the Rayyan platform. This enables semi-automatic detection of duplicates and facilitates conflict resolution and selection organization (Ouzzani et al., 2016). If there was disagreement on specific cases, the blind review was removed and the cases were discussed. If consensus could not be reached, a third evaluator was consulted to help make the final decision. After the initial identification of studies using the search strategy, a second detailed screening was performed. Abstracts were then reviewed to identify and exclude studies that did not meet the inclusion criteria. The texts were then carefully evaluated.

### 2.4 Quality and bias risk assessment

Two authors independently assessed the quality and risk of bias of each study's methodological aspects using a 13-point checklist adapted from previous neuroimaging meta-analyses (Zhao et al., 2022; Zhou et al., 2024). In addition to demographic and clinical characteristics, this checklist covers key variables in rs-fMRI studies. Each criterion was scored as follows: 1, fully met, 0.5 for partially met; and 0, unfulfilled. We included studies with scores of >9 in the review (**Figures 5** and **6**). In cases of disagreement, differences in interpretation were discussed between the authors, with the involvement of a third reviewer if necessary.

Details regarding the rs-fMRI methodology (i.e., image acquisition parameters, quality control of head motion, multiple comparison corrections, and covariates) are presented in the Supplementary Tables A and B.

### 2.5 Data synthesis methods

This section presents a descriptive summary of the results. The heterogeneity of the seeds in the included studies prevented us from conducting a meta-analysis using Seed-Based D Mapping (Müller et al., 2018). The following sociodemographic and clinical characteristics were extracted: sample size, age, gender, diagnostic method, handedness, illness duration, depressive state/episode, symptom severity, and medication status. This information and the key results on alterations in rs-FC from seed-to-whole-brain analysis (FM vs. HC/MDD vs. HC) were extracted. Weighted means and standard deviations were used for continuous variables extracted and cited above. Continuous variables were compared between the groups using the *t-*test for parametric data for descriptive analysis.

## 3 Results

**Figure 1:**
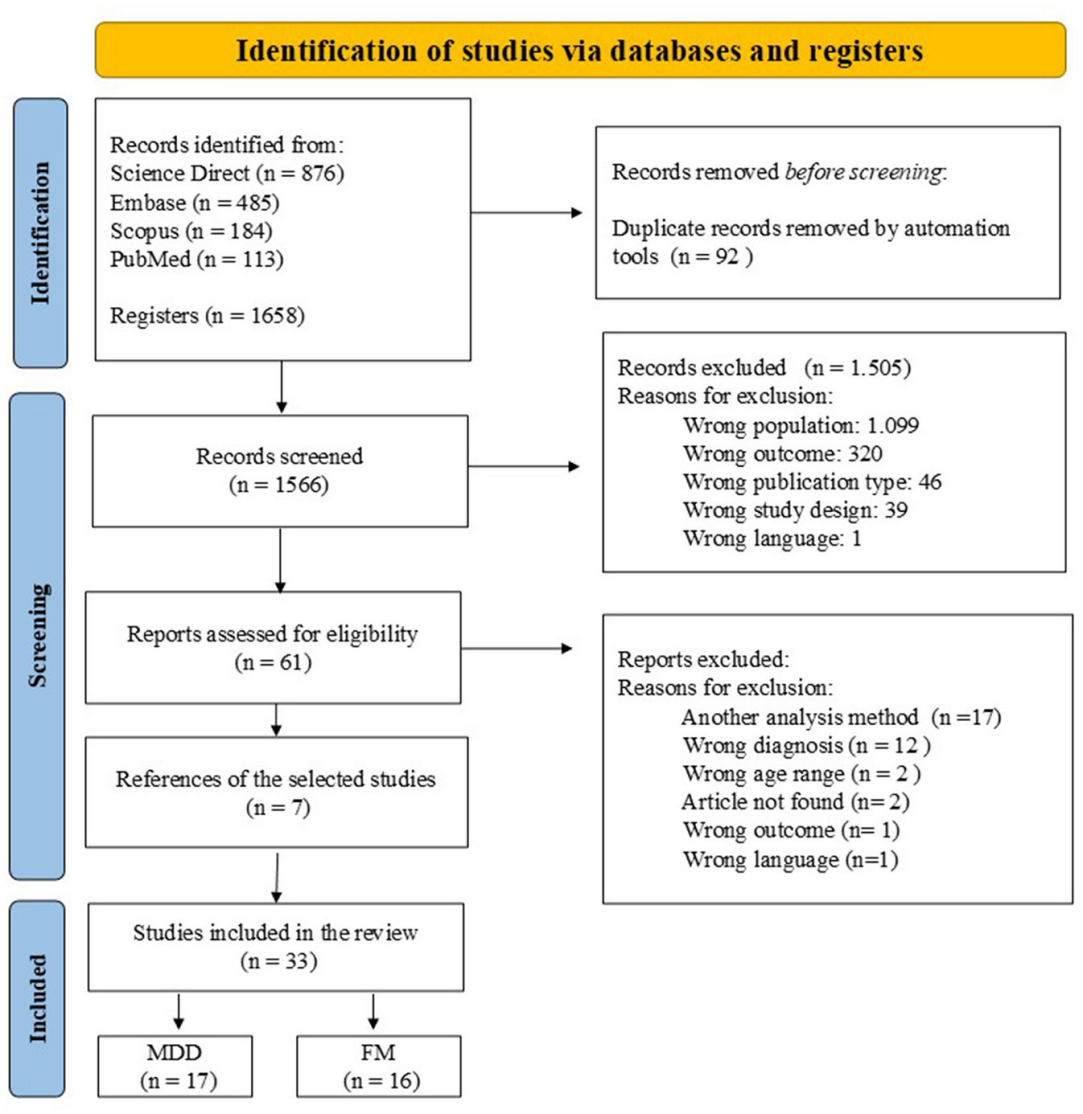
PRISMA flowchart for systematic review.

The search strategy is summarized in Figure 1 as indicated in the PRISMA flowchart. Our search strategy initially identified 1,658 potential articles, of which 26 met the inclusion criteria for this review. Seven eligible studies were identified through references within the selected articles, resulting in a total of 33 studies. The pooled sample comprised a total of 1,877 individuals, including 947 patients and 930 controls, with 1,087 females (57.9%) and 790 males (42.1%) (1.38:1). Among patients, 553 (58.4%) of the patients were female and 394 (41.6%) were male (1.4:1). Among the controls, 534 (57.4%) were female and 396 (42.6%) were male (1.35:1). The mean patient had an average age was 39.83 years.

### 3.1 Demographic characteristics of MDD patients

The systematic review, which included patients with MDD, comprised 17 studies with 573 patients and 556 control subjects. Almost half of the sample were women (57.9%), and the mean age of patients with depression was 36.11 years (SD = 9.87). In terms of clinical characteristics, 299 patients reported experiencing their first depressive episode and 283 were drug naive. In addition, 171 patients had recurrent MDD and one study included a sample of patients with treatment-resistant depression. The duration of depression ranged from 2 months to 6 years, with four studies reporting a duration of more than 2 years. The Hamilton Depression Rating Scale (HDRS) was used to assess the severity of depressive symptoms in our sample. The mean score was 23.53 (SD = 4.85), indicating moderate depressive symptoms.

### 3.2 Demographic characteristics of fibromyalgia patients

We included 16 studies that assessed FC in patients with FM. The sample consisted of 374 FM patients with a mean age of 45.5 years (SD = 10) and 374 healthy controls (mean age 46 ± 9.8 years), with a predominance of women (98.1%). FM diagnosis was based on the American College of Rheumatology (ACR) criteria in 88.5% of the studies (*n* = 331): half of the studies used the ACR-1990 criteria (Wolfe et al., 1990), while the remaining used the ACR-2010 (Wolfe, 2010) (*n* = 130) or 2016 criteria (Wolfe et al., 2016) (*n* = 26). The sample had an average Fibromyalgia Impact Questionnaire (FIQ) score of 54.8 (SD = 14), revealing a moderate impact of FM symptoms on quality of life. The severity of depressive symptoms was assessed using two different scales: the Beck Depression Inventory-II (BDI-II) was used in seven studies (*n* = 143), with a score of 19 (SD = 10.2), indicating moderate depressive symptoms. In contrast, the Hospital Anxiety and Depression Scale—Depression subscale (HADS-D), used in six studies (*n* = 177), indicated mild depressive symptoms (6.58 ± 3.9).

The most common medications used by patients included analgesics, muscle relaxants, non-steroidal anti-inflammatory drugs (NSAIDs), antidepressants, and anticonvulsants. Table 1 presents further details on the sample characteristics of MDD and FM.

**Table 1:** Demographic and clinical characteristics of MDD and fibromyalgia.

	**MDD**	***FM***	***p*-value**
	***n =* 573**	***n =* 374**	
	**Mean ±SD**	**Mean ±SD**	
Age (years)	36.11 ± 9.87	45.53 ± 10.2	< 0.001
Age range (years)	18–75	18–75	
Mean condition duration (months)	28.16 ±32.4	142.6 ± 80,7	< 0.001
Handedness (left/right)	9/384	3/136	
Gender (male/female)	241/332	7/367	
Gender female (%)	57.9	98.1	
*Dx tool—FM*	*n* (%)	*n* (%)	
American College of Rheumatology (1990)	–	175 (46.79)	
American College of Rheumatology (2010)	–	130 (34.76)	
American College of Rheumatology (2016)	–	26 (6.95)	
N/I	–	43 (11.5)	
*Dx tool—MDD*	*n* (%)	*n* (%)	
MINI + DSM IV	56 (9.8)	–	
ICD-10	34 (5.9)	–	
DSM-IV	483 (84.3)	–	
	**Mean** ***±*****SD**	**Mean** ***±*****SD**	
Hamilton Depression Rating Scale, score	23.53± 4.8	–	
Hospital Anxiety and Depression Scale, score	–	6.58 ±3.9	
Beck Depression Inventory-II, score	–	19 ±10.16	
Fibromyalgia Impact Questionnaire, score	–	54.82 ± 14.89	
Visual Analog Scale (0–10)	–	5.19± 2.04	
*Medication use*	*n*	*n*	
Antipsychotics	4	–	
Antidepressants	146	105	
NSADIs	–	64	
Anxiolytics	4	31	
Analgesic drugs^*^	–	19	
Muscle relaxants	–	32	
Anticonvulsants	4	30	
Hypnotics	5	–	
Opioids	–	5	
NSAID, pain, and sleep medication¢	–	68	
Drug-naive	283	–	
No Medication	110	20	
N/I	–	94	

Data are presented as mean ±SD or n (%). FM, Fibromyalgia Patients; MDD, Major depressive disorder; FIQ, Fibromyalgia Impact Questionnaire; NSAIDs, non-steroidal anti-inflammatory drugs; ICD-10, International Classification of Diseases; DSM, Diagnostic and Statistical Manual of Mental Disorders. N/I, not informed; SD, Standard deviation. ^*^Analgesic drugs include ibuprofen and, paracetamol. ¢Combined medications. Healthy controls of MDD (n = 556): age mean 34.6 ± 10.1. Healthy controls of FM (n = 374): age mean 46.0 ± 9.8. Test t—Unpaired test.

### 3.3 Major findings of seed-based functional connectivity

Among the 33 articles included in this study, a total of 25 seeds were found for the analysis of whole-brain FC. For better comprehension, we chose to group the seeds according to functional networks in the following areas (Bednarska, 2019): (1) *reward network* (RN): ventral tegmental area (VTA), nucleus accumbens (NAcc), striatum, caudate nucleus, habenula; (2) DMN: posterior cingulate cortex (PCC), precuneus, mPFC, superior temporal gyrus (STG), supramarginal gyrus (SMgyr) and hippocampus; (3) *salience network* (SN): insula, mid cingulate cortex (MCC), amygdala, ACC; (4) *central executive network* (CEN): inferior parietal lobe (IPL), intraparietal sulcus (IPS), DLPFC, inferior frontal gyrus (IFG), and middle frontal gyrus (MFG); (5) *sensorimotor network* (SMN): globus pallidus (GP), the primary motor cortex (M1), primary and secondary somatosensory cortices (SI/SII), parietal operculum, thalamus, and supplementary motor areas (SMA); (6) *central autonomic network* (CAN): hypothalamus, PAG. One study was also found in the literature search that specifically reported findings in the visual network. However, due to their high level of specificity, they were not included in the pooled analyses.

The articles observed high heterogeneity regarding seed choice, highlighting the complexity of the symptoms in the two clinical conditions and their relationship with brain targets. More details regarding the sample characteristics, clinical variables, and major findings of FC from each study are presented in in the Supplementary Tables C and D.

#### 3.3.1 Seed-based analysis of functional connectivity in MDD

In the analysis of 17 MDD studies, 13 seed regions were selected to examine whole-brain FC. The most frequently investigated seed regions included the insula, the ACC, and the precuneus. Among these, SN, DMN, and RN seeds were the most studied in MDD-related research. We found that seeds from networks involved in attention and emotion regulation—specifically, the left insula and the subgenual ACC (sgACC) within the SN—as well as those related to internal processes (DMN seeds), often exhibited reduced connectivity with their connected regions.

The left insula showed decreased rs-FC with its contralateral MFG, superior frontal gyrus (SFG), and middle occipital gyrus (mOccipital Gyr), indicating diminished interhemispheric communication between the insula and these brain regions in MDD patients compared to HC. Also, decreased rs-FC was observed between the left insula and right ACC. The bilateral ACC seed showed decreased FC with several brain regions, including the hippocampus, PCC, angular gyrus, thalamus, striatum, insula, STG, middle temporal gyrus (MTG), SFG, and cerebellum. Additionally, decreased connectivity was observed between the left dorsal ACC and the bilateral MFG. In contrast, increased connectivity was found between the left dorsal ACC, the left precentral gyrus, and the left angular gyrus.

Increased connectivity was observed between the right insula, left thalamus, and habenula. The left insula showed augmented rs-FC with the left IFG, amygdala, and superior temporal sulcus (STS). In MDD patients, an increase in the connectivity of the amygdala seed with the IFG, right MFG, right MCC, and paracingulate gyrus regions was also observed.

**Figure 2:**
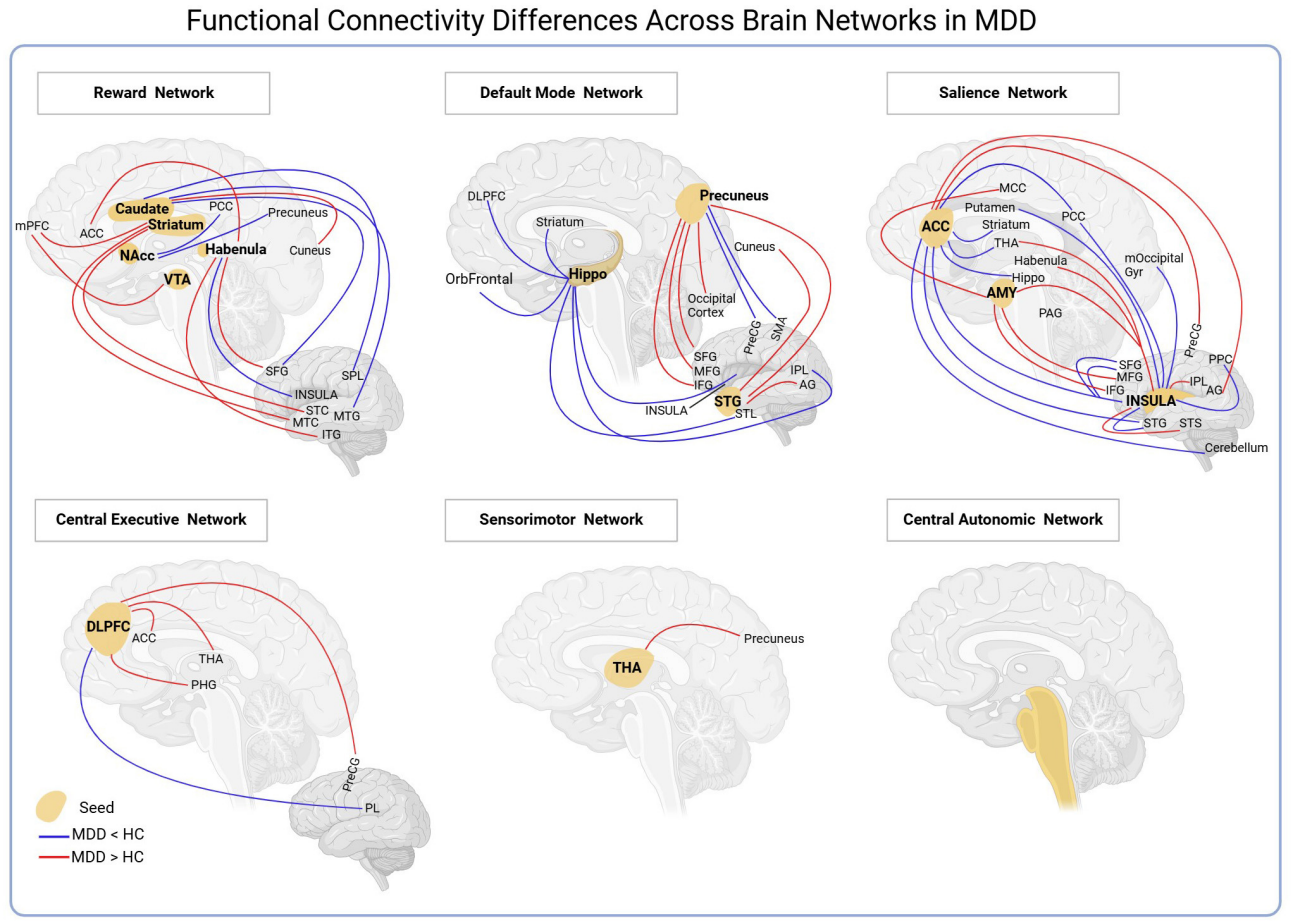
The seeds evaluated in the MDD studies were grouped into six networks. The most expressive networks were reward, default mode, and salience networks. See the Abbreviations section for definitions.

Precuneus showed a reduction in FC with motor areas, such as the bilateral fusiform gyrus, the right SMA, and the left precentral and postcentral gyrus. It showed increased FC with executive function regions, including the right superior, MFG, and left middle/inferior frontal gyrus. Figure 2 illustrates the FC differences between MDD and HC.

#### 3.3.2 Seed-based analysis of functional connectivity in fibromyalgia

**Figure 3:**
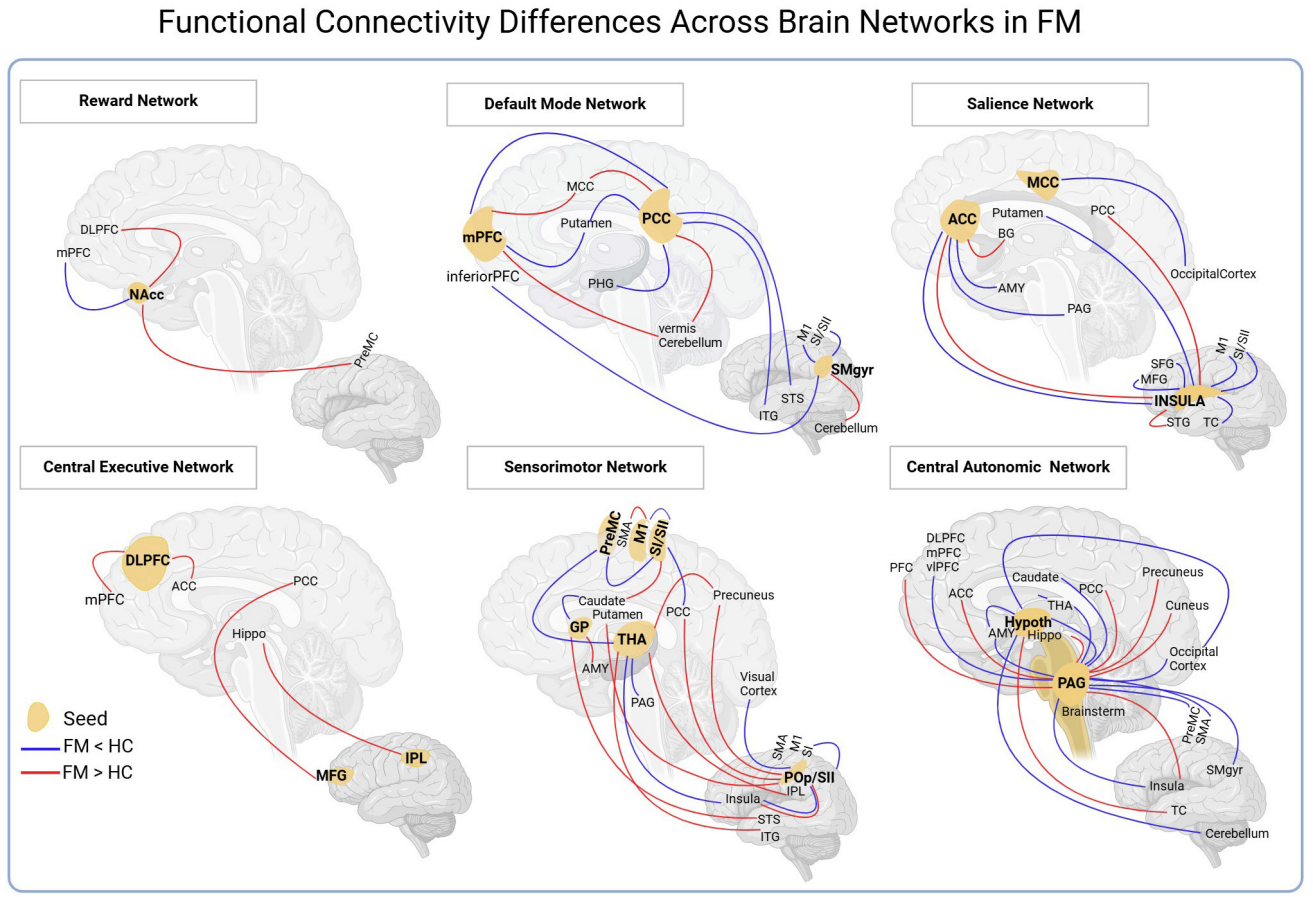
The seeds evaluated in the fibromyalgia studies were grouped into six networks. The most expressive networks were sensorimotor and central autonomic. See the Abbreviations section for definitions.

Several seed regions were selected to analyze brain connectivity. The seeds most mentioned in the 16 studies with FM patients were the PAG, insula, parietal operculum/secondary somatosensory cortex (SII), PCC, thalamus, mPFC, IPL, and primary somatosensory cortices, among others. The main findings are presented in Figure 3.

In studies that investigated the FC of FM patients in rs-fMRI, we observed that the seeds from the CEN group exhibited more results with increased FC. Based on these studies, FM patients, compared to controls, showed an increased FC in all seeds of the CEN group (IPL, DLPFC, MFG) for regions of the PCC, ACC, hippocampus, and mPFC. However, most seeds from the CAN and SN groups demonstrated reduced FC. CAN seeds showed reduced connectivity with SMA, amygdala, thalamus, insula, PCC, and PFC (dl, dm, and vl). While the SN seeds exhibited low connectivity with the SI/M1, MFG, SFG, putamen, and ACC regions in FM patients.

Our findings indicated that numerous seeds (e.g., insula, PAG, PCC, SI/SII cortex, SMgyr, and thalamus) showed reduced FC to sensorimotor areas in patients with FM. We identified that the PAG, insula, and parietal operculum/SII seeds are the most investigated in studies with rs-fMRI in patients with FM. The PAG seed showed FC reduction behavior for regions involved with the perception and modulation of pain in patients with FM, such as the insula, amygdala, thalamus, SMA, DLPFC, and dmPFC. This reduction showed correlations with clinical aspects, such as pain intensity, quality of life, catastrophizing, and duration of the disease.

Similarly, the insula seed has reduced connectivity with pain processing areas (SI, M1, MFG, SFG, ACC, and putamen), influencing how the pain is interpreted and responded to.

#### 3.3.3 Seed-based analysis of shared connectivity pathways between FM and MDD

**Figure 4:**
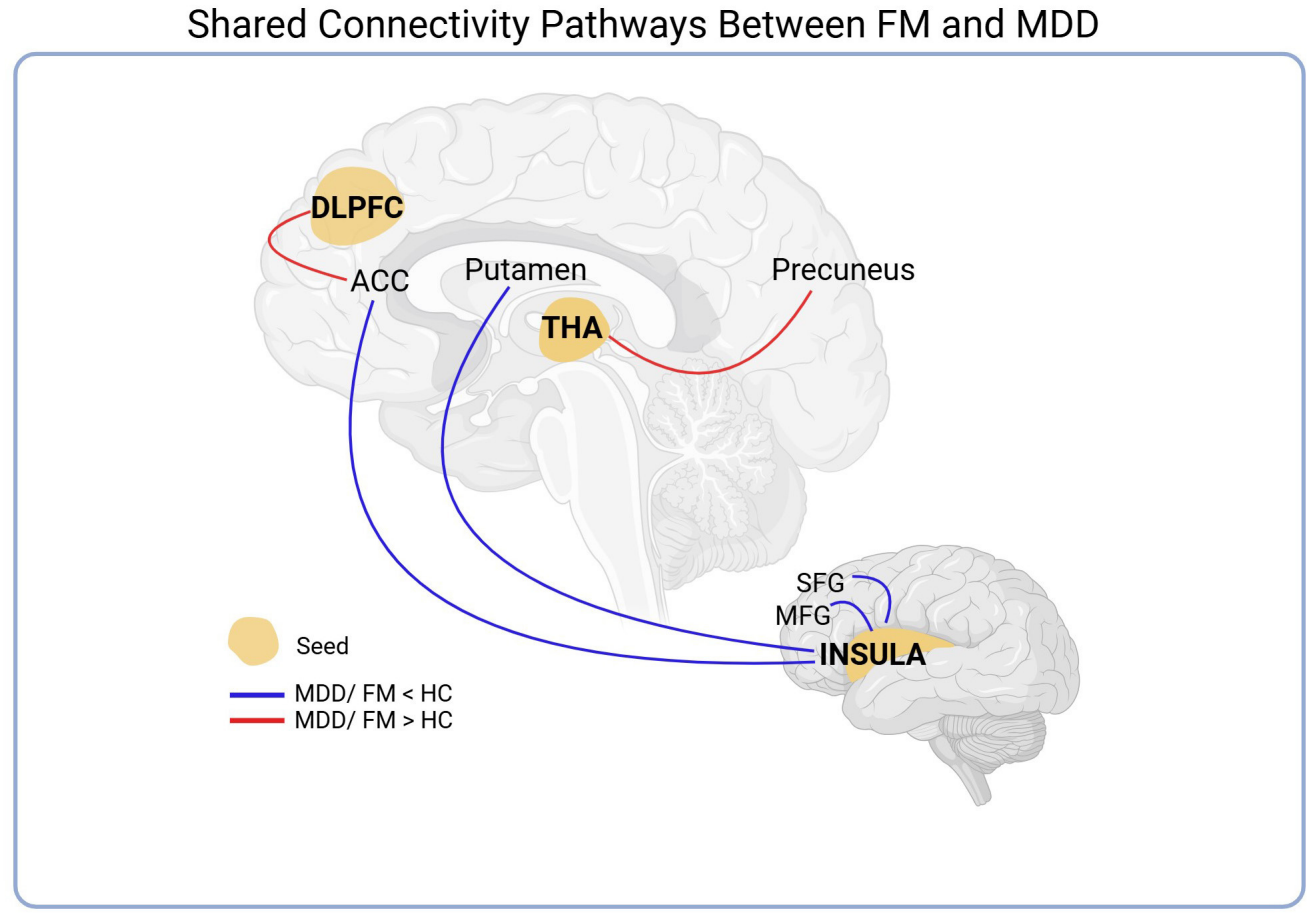
Seed-based analysis of shared connectivity pathways between FM and MDD. The DLPFC and thalamus seeds exhibited increased connectivity to the ACC and Precuneus, respectively, in both groups. Additionally, a reduction in the functional connectivity of the seed insula with the MFG, SFG, ACC, and putamen was also observed. See the Abbreviations section for definitions.

A similar behavior of FC was observed between the two clinical conditions—FM and MDD, found in ten of the selected articles (Figure 4). Analyzing studies with rs-fMRI in patients with depression and with fibromyalgia, it was possible to verify that the DLPFC seed exhibited increased connectivity to ACC in both groups. Furthermore, the FC between the thalamus and precuneus increased in both depressed and FM patients.

In addition to this result, we also observed a reduction in the FC of the seed insula with the areas involved in executive functions (MFG and SFG) and regulatory actions on attention and emotional response to stimuli (ACC) and with regions related to motor control and movement coordination (putamen).

### 3.4 Relationship between FC alterations and clinical symptoms of FM and MDD

In addition to comparing rs-FC between patients and controls, some studies were able to demonstrate a correlation between connectivity alterations and symptom severity. Ceko et al. (2013) found, in young FM women, a negative correlation between the reduction in FC between the left anterior insula and the ACC with levels of catastrophizing thinking and pain intensity; in other words, this decrease in connectivity in FM is associated with a worsening of symptoms and disease severity (Ceko et al., 2013). Similar results were found in two other studies with depressed patients. In Wang et al. (2018), decreased left insula-ACC connectivity showed a negative correlation with HDRS scores (*r* = −0.5841, *p* = 0.0034) (Wang et al., 2018). Likewise, in Yue et al. (2024), this decrease in FC was also negatively correlated with the severity of depressive symptoms, assessed by HDRS (*r* = −0.3375, *p* = 0.025) (Yue et al., 2024).

### 3.5 Bias risk assessment

**Figure 5:**
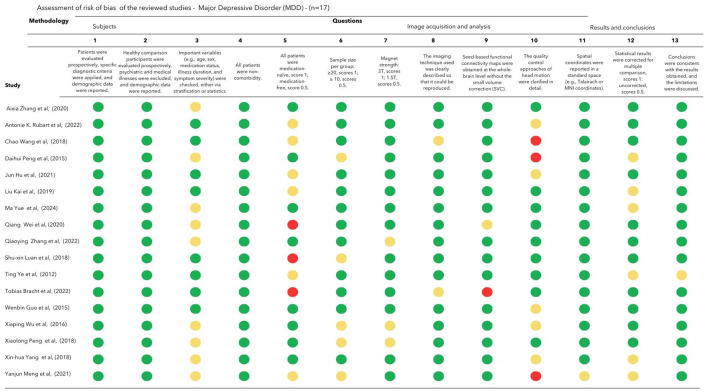
Assessment of risk of bias of the reviewed MDD studies (*n* = 17). Each criterion is scored as follows: 1 for fully met (**green mark**), 0.5 for partially met (**yellow mark**), and 0 for unfulfilled criteria (**red mark**).

**Figure 6:**
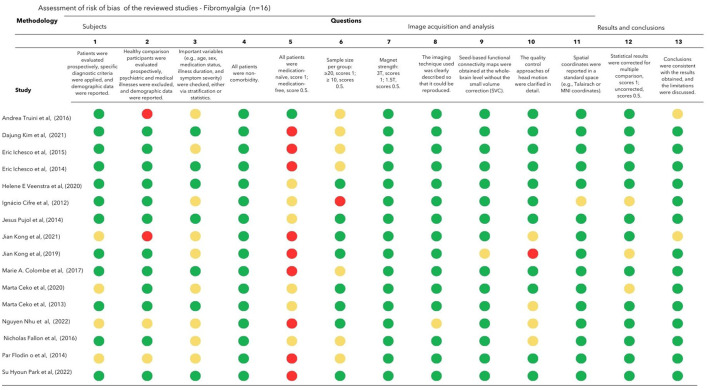
Assessment of risk of bias of the reviewed fibromyalgia studies (*n* = 16). Each criterion is scored as follows: 1 for fully met (**green mark**), 0.5 for partially met (**yellow mark**), and 0 for unfulfilled criteria (**red mark**).

The risk of bias, evaluated by the fMRI checklist of the 33 studies included in this review, is presented in Figures 5 and 6. All included studies met the cutoff point of 9. The main limitations of FM studies are related to small sample sizes and the lack of clear descriptions of important clinical variables (eg, age range, laterality, and comprehensive symptom assessment scales). None of the recruited participants were described as drug-naive, and several were on central nervous system medications. Antidepressants, analgesics, mood stabilizers, and antipsychotics affect and modulate resting-state networks (Posner et al., 2013; Wang et al., 2017; Altinay et al., 2018). However, patients with FM often need multiple medications for their symptoms and comorbidities. Vincent et al. (2016) found that 40% of patients took three or more medications to manage FM symptoms. The differences in ACR's revised version of the diagnosis were also noticed. Seven studies used the ACR-1990 which focused mainly on tender points (Wolfe et al., 1990), and the other half with the revised versions focused on widespread pain and symptom severity (Wolfe, 2010).

The studies involving patients with depression included a larger sample size compared to those on FM, with only four studies having a sample size of 20 or fewer participants (Peng et al., 2015, 2018; Wu et al., 2016; Luan et al., 2019). In general, the fMRI methodology and outcome measure were well described in FM and MDD studies.

## 4 Discussion

Understanding the neural mechanisms underlying FM and major depressive disorder MDD is crucial for identifying shared and distinct pathophysiological pathways that contribute to chronic pain and affective dysregulation. Both conditions exhibit high comorbidity, with overlapping clinical symptoms such as heightened pain sensitivity, cognitive dysfunction, fatigue, and mood disturbances. Despite these similarities, FM and MDD also present unique neurophysiological features, suggesting that while they share common alterations in rs-FC, they also involve disorder-specific disruptions. This review systematically examined rs-FC alterations from seed-based fMRI studies, mapping the neural signatures of FM and MDD across different brain networks involved in pain perception, emotion regulation, and cognitive control.

### 4.1 Shared functional connectivity alterations in FM and MDD

Reduced rs-FC between the left insula and the ACC was consistently associated with worsening clinical symptoms. Lower connectivity between these regions correlated with greater catastrophizing and higher pain intensity in individuals with FM, as well as increased severity of depressive symptoms in patients with MDD. The insula-ACC circuit plays a central role in modulating both affective and sensory processing, suggesting an association between the clinical profiles of both conditions (Ceko et al., 2013; Wang et al., 2018; Yue et al., 2024). This connection likely functions as an input-output system, integrating self-awareness with cognitive, affective, and physical states (Medford and Critchley, 2010). By processing interoceptive signals in the insula and re-representing them in the ACC, this system facilitates dynamic regulation of internal states, supporting adaptive responses to internal and external stimuli. These mechanisms are closely linked to top-down modulation of pain, as the ACC and insula are central components of the medial pain system, receiving inputs from the midline and intralaminar nuclei of the thalamus (Vogt, 2005). A reduction in FC between these regions in FM and MDD may impair the ability to accurately perceive and respond to internal bodily states and external events (Medford and Critchley, 2010), contributing to dysregulated psychological and sensory experiences in both conditions. Such disruptions are likely to exacerbate symptoms, including pain, emotional distress, and cognitive impairment (Barthas et al., 2015; Yan et al., 2024).

Beyond the ACC, the insula also shows a reduced rs-FC with the MFG and SFG in both FM and MDD. These regions are essential for the cognitive-evaluative dimension of pain processing (Ichesco et al., 2014; Ceko et al., 2013) and significantly influence the perception and regulation of pain. Disruptions in these connections may result in sensory processing dysregulation, with hyperactivity in the insula overwhelming frontal areas, leading to poor regulation of sensory stimuli. Over time, chronic pain can exacerbate this dysfunction, triggering emotional and cognitive alterations that further intensify the pain experience. Supporting this idea, Tian et al. (2021) found decreased rs-FC in the MFG and SFG in patients with chronic migraines compared to HC. Our findings align with these results, emphasizing a common neural alteration underlying pain-related disorder.

Our review also found reduced rs-FC between the insula and putamen in FM and MDD patients. These brain regions are closely associated with affective processing and sensorimotor functions, both of which are frequently implicated in chronic pain and depression (Haber and Knutson, 2009). The putamen plays a key role in the selection, planning, and execution of motor behaviors and is also involved in reward-related processes (Crutcher and DeLong, 1984; Jankowski et al., 2009). Additionally, it has been associated with the experience of sadness (Lindquist et al., 2012) and linked to fatigue (Nakagawa et al., 2016). The posterior putamen is strongly connected to sensorimotor functions, including its affective dimensions (Pauli et al., 2016). The insula, a key player in this scenario, integrates sensory and emotional information, projecting to the ventral striatum, including the putamen, which is critical for processing visceral and emotional signals (Fudge et al., 2005). The disrupted insula-putamen connectivity may contribute to the overlapping affective and sensorimotor disturbances observed in FM and depression.

### 4.2 Distinct connectivity alterations and their clinical implications

Increased rs-FC between the DLPFC and ACC in MDD may reflect heightened neural responses to negative stimuli, as well as disrupted error monitoring and executive control within the frontal-cingulate network (Schlösser et al., 2008). This heightened connectivity could represent a compensatory mechanism for impaired self-regulation and sensory processing, potentially aligning with the generalized hypervigilance hypothesis in FM (McDermid et al., 1996; Borg et al., 2018). This mechanism may explain the contrasting rs-FC patterns observed: (a) The insular-ACC network showed reduced connectivity, suggesting impaired affective and sensory integration. (b) The DLPFC-ACC network exhibited increased connectivity, potentially as an adaptive response aimed at enhancing executive control and regulation. Evidence from animal models of chronic pain indicates that prolonged pain results in hyperexcitability between the ACC and PFC (Lee et al., 2022). Acute pain stimuli activate the PFC (Ong et al., 2018), while intense noxious stimuli engage regions such as the ACC, PFC, and DLPFC, situating pain within a cognitive-evaluative framework (Nir et al., 2008). A resting-state electroencephalography (EEG) study in women with FM (Alves et al., 2023) reported heightened beta-3 frequency band connectivity between the left DLPFC and right ACC compared to controls. These findings, observed across multiple methodologies, strengthen the case for the involvement of these neural mechanisms in FM.

Furthermore, increased FC between the thalamus and precuneus was observed in MDD and FM compared to HC. The precuneus is a central hub of the DMN (Raichle and Raichle, 2001; Zhang et al., 2012), while the thalamus, though not traditionally part of the DMN, exhibits strong rs-FC with the precuneus (Tomasi and Volkow, 2011; Cunningham et al., 2017). The DMN is involved in self-referential thoughts and mind wandering, acting as an internal narrative system (Raichle, 2015; Menon, 2023). Increased FC between these regions may reflect depressive biases and rumination, reducing external engagement (Chen et al., 2020). In FM, this heightened thalamus-precuneus connectivity could relate to pain catastrophizing (Ellingsen et al., 2021).

### 4.3 Methodological considerations and future directions

The scientific advancements in neuroscience have significantly improved our understanding of the mechanistic underpinnings of FM and MDD. Investigating rs-FC through advanced neuroimaging techniques is essential for elucidating their neurobiological mechanisms. The neuroimaging systematic review and meta-analysis by Cavicchioli et al. (2025), which specifically examines FM, offers important contributions into the pain matrix. Despite these advances, the integration of rs-FC with complementary modalities, such as positron emission tomography (PET) and (EEG), allows for a more comprehensive analysis of real-time brain dynamics. The combined use of fMRI and EEG offers a complementary approach for identifying biomarkers in FM and MDD, leveraging the spatial precision of fMRI with the high temporal resolution of EEG. While fMRI maps alterations in functional connectivity within key networks, such as the descending pain modulatory system (DPMS), DMN, and SN (Cifre et al., 2012; Apkarian et al., 2005; Ceko et al., 2020), EEG captures oscillatory activity changes associated with sensory processing, cognitive dysfunction, and emotional regulation (Keune et al., 2011).

In FM, fMRI studies reveal altered connectivity in pain-related networks, including the PAG, ACC, and insula (Ossipov et al., 2010; Staud, 2009). Meanwhile, EEG studies show increased theta activity and reduced alpha activity, suggesting central sensitization (Alves et al., 2023). In MDD, fMRI highlights hyperactivity in DMN and reduced connectivity in executive control regions (Gollan et al., 2014), while EEG identifies frontal alpha asymmetry, which correlates with emotional dysregulation and cognitive impairment (Gkintoni et al., 2025). The combination of these modalities may enhance diagnostic precision and treatment monitoring, as fMRI assesses long-term connectivity changes, while EEG provides temporal dynamics of alpha asymmetry, which might be a relevant phenotype in the examined patient population (Keune et al., 2011).

Identifying shared and distinct neurosignatures may aid in developing biomarkers for symptom severity, disease progression, and treatment response. To improve reliability and comparability, rigorous methodological standards must be adopted. The 2016 ACR criteria for FM, incorporating pain, depression, sleep disturbances, and cognitive impairment, provide a structured framework for patient characterization. However, integrating rs-FC data with clinical and psychophysical measures is essential for establishing clearer links between neural dysfunctions and symptom severity. Systematic reporting of comorbidities and medication effects is also crucial for refining study populations and improving connectivity analyses. FM is primarily associated with alterations in the PAG and motor areas (Truini et al., 2016), while MDD involves dysfunctions in the SN and DMN. (Kaiser et al., 2015). As the PAG is a central structure of the DPMS (Coulombe et al., 2016, 2017), its dysfunction may contribute to chronic and treatment-resistant pain. fMRI studies show that the ventrolateral PAG has indirect connections with central lateral and medial pain pathways, the ACC, and the upper pons/medulla (Coulombe et al., 2016), modulating pain through descending projections to spinal dorsal horn neurons (Ossipov et al., 2010). Therefore, the integrating rs-FC data with DPMS function and clinical measures may enhance our understanding of pain modulation in FM and MDD. rs-FC between DPMS structures, particularly M1 and the ventral lateral thalamus, correlates with pain modulation capacity and tDCS response in FM (Cummiford et al., 2016). Additionally, motor cortex stimulation restores thalamic function in chronic pain disorders, highlighting a potential neuromodulatory target (Tsubokawa et al., 1993). Furthermore, DPMS dysfunction presents distinct patterns in FM and MDD, correlating with symptom severity and functional impairment (Cardinal et al., 2019; Soldatelli et al., 2021).

However, at date most studies remain cross-sectional, limiting the ability to determine whether rs-FC alterations in DPMS and related networks are primary pathophysiological mechanisms or secondary adaptations to chronic pain. Another key uncertainty is whether rs-FC changes in FM and MDD remain stable over time or exhibit dynamic fluctuations during disease progression. Addressing these gaps through longitudinal research is critical for defining rs-FC as a biomarker and identifying predictors of treatment response. Future research should focus on integrating rs-FC biomarkers with clinical and psychophysical assessments to improve diagnostic precision and personalized therapeutic strategies. Combining rs-FC evaluation of DPMS function with pain severity and functional impact measures could refine neuromodulatory and pharmacological interventions. Bridging neuroimaging research with clinical practice may lead to more effective, individualized treatments for FM and MDD, ensuring that biomarkers translate into tangible improvements in patient care.

### 4.4 Limitations

Although this review provides valuable insights into the neural mechanisms underlying FM and MDD, several methodological considerations must be addressed. *First*, one of the primary limitations in seed-based rs-FC studies is the priori selection of regions of interest (ROIs). This approach may introduce confirmation bias by restricting analyses to predefined networks while potentially overlooking novel connectivity patterns ( Wu et al., 2018a,b). *Second*, variability in fMRI acquisition parameters—such as scan duration, spatial resolution, and preprocessing pipelines—can affect the reproducibility of rs-FC findings (Birn et al., 2013). These inconsistencies highlight the need for a more strategic and coordinated approach in study design, data collection, and statistical analyses to enhance replicability and facilitate cross-study harmonization. To address these methodological challenges, we suggest that future research should: (1) employ standardized, atlas-based ROIs—ideally comparing multiple seed definitions across different atlases—to enhance reproducibility and cross-study harmonization; (2) integrate hypothesis-driven seed-based methods with data-driven techniques to balance targeted and exploratory analyses; (3) provide detailed methodological reporting, including seed coordinates, size, and selection rationale, to improve transparency; and (4) establish standardized fMRI acquisition parameters (e.g., scan duration, repetition time) in study designs, following recommendations such as those from the American Journal of Neuroradiology (Kumar et al., 2024). *Third*, studies on MDD predominantly included drug-naive patients, whereas FM studies involved participants taking various medications, such as antidepressants, anticonvulsants, and non-opioid analgesics. However, our findings remained consistent regardless of illness duration or medication status between the two populations. This underscores the relevance of MDD-FM comorbidity as a factor contributing to greater symptom burden and chronicity, which are often linked to poorer clinical outcomes. *Fourth*, the assessment of depression varied across studies due to differences in rating scales, leading to potential interpretation biases. To improve consistency, we recommend that future FM studies adopt the HDRS for standardization, as it is widely used in MDD research to assess treatment efficacy and is recognized for its validity and sensitivity (Bagby et al., 2004; Faries et al., 2000). *Fifth*, a more strategic and coordinated approach aligned with the biopsychosocial model could enhance chronic pain management. This approach should inspire future studies to focus on elucidating the underlying mechanisms of pain to enable more personalized treatments. Additionally, prioritizing research that integrates this model and employs validated functional measures will improve the assessment of pain's impact on patients' lives. Guidelines such as IMPACT emphasize the importance of standardization in evaluating pain and functionality (Carey et al., 2020). Accordingly, we recommend using the Numeric Pain Scale (NPS) ranging from 0 to 10, along with functional measures assessing quality of life, social participation, and physical performance. Lastly, in fMRI studies, ensuring sample consistency regarding laterality is crucial to enhancing the reproducibility and reliability of findings. Standardized methodologies in data acquisition and analysis will further strengthen the robustness of future research in this field.

## 5 Conclusion

Our review identifies a potential neurosignature shared by MDD and FM, highlighting the salience network as a common neural pathway involved in both conditions. Key areas of dysfunction include the insula, anterior cingulate cortex, and prefrontal regions, which are crucial for processing both pain and emotions. In FM, altered functional connectivity was observed in the periaqueductal gray and sensorimotor areas, reflecting impairments in pain modulation. In MDD, disruptions primarily affect the salience network and default mode network, contributing to emotional dysregulation and negative cognitive bias. These findings suggest an overlapping neural mechanism between emotional processing and pain perception, reinforcing their bidirectional influence. Given the high comorbidity between FM and MDD, understanding this interplay is crucial for developing personalized treatment strategies that address both pain symptoms and emotional dysregulation effectively.

## Data Availability

The original contributions presented in the study are included in the article/Supplementary material, further inquiries can be directed to the corresponding author.
