# Supplementary material for: Seed-based resting-state connectivity as a neurosignature in fibromyalgia and depression: a narrative systematic review
Source: Front Hum Neurosci. 2025 Apr 28;19:1548617. doi: 10.3389/fnhum.2025.1548617 (PMC12066659; doi:10.3389/fnhum.2025.1548617)
Supplement: Supplementary file 1 [file Table_1.docx]

Table A: the details regarding the rs-fMRI methodology in the MDD studies (i.e. image acquisition parameters, quality control of head motion, multiple comparisons corrections, and covariates).

| **Study** | **Pre-processing and processing Software packages** | **Smoothing kernel size** | **Statistical covariates** | **Correction for multiple comparison** | **Scan duration/ volume no.** | **Repetition time** | **Voxel size** | **Eyes open/ closed** |
| --- | --- | --- | --- | --- | --- | --- | --- | --- |
| Aixia Zhang et al, 2020 | DPARSF & SPM | 6-mm | Global mean signal, head motion parameters,cerebrospinal fluid signal and white matter | Corrected for multiple comparisons using Monte Carlo simulations | 7min | 2s | 3×3×3 mm | N/I |
| Antonie K Rubart et al, 2022 | CONN | 8-mm | Study center | Cluster-defining primary threshold to p < 0.001 and employed an FWE-corrected threshold of p < 0.05 at cluster-level. Bonferroni correction for the number of seeds (0.05/12) resulted in a significance level of 0.004. | 8min | 2s | 3×3x3mm | closed |
| Chao Wang et al, 2018 | DPARSF | 6-mm | Age, gender, education, motion parameters, white matter, and cerebrospinal fluid signals | Cluster-level Monte Carlo simulation (5000 times) corrected threshold of p < 0.05 (cluster-forming threshold at voxel-level p < 0.001). | 8min | 2s | 3×3×3 mm | closed |
| Daihui Peng et al, 2015 | SPM | 8-mm | Age, gender, education, motion parameters, white matter, and cerebrospinal fluid signals | FWE cluster-level corrected p value <0.05 | 5min | 3s | 3.75x3.75x5mm | closed |
| Jun Hu et al, 2021 | DPARSF, SPM  & DPABI | 8mm | Age, gender, education, and mean FD values | FWE cluster-level corrected p value <0.05 and voxel-level uncorrected at p < 0.001. Correlation coefficients below 0.05 according to the FDR-adjusted p-values were retained as statistically significant. | 8min | 2s | 3×3×3 mm | closed |
| Liu Kai et al, 2019 | SPM | 8-mm | Age, gender, education, BMI, daily cigarette use, brain volume, mean FD , and episode of illness | Bonferroni correction for the number of clusters p>0.05 | 8min | 2s | 3.4×3.4×3.4 mm | N/I |
| Ma Yue et al, 2024 | DPABI & SPM | 6-mm | Age, gender, education, and mean FD | Gaussian random field correction (GRF), combined voxel-wise P-value < 0.005 with cluster P-value < 0.05 (two-tailed) set a threshold | N/I | 2s | 3×3×3 mm | N/I |
| Qiang Wei et al, 2020 | DPAB & SPM | 4-mm | Age, gender, education, grey matter, FD power | Cluster-level FD-corrected threshold of p < 0.05 (cluster-forming threshold at voxel-level p < 0.001). | 8min | 2s | 3×3×3 mm | closed |
| Qiaoying Zhang et al, 2022 | AFNI & FSL | 6-mm | N/I | FDR-corrected threshold of p < .05. The cluster threshold was 0.001, and 10 000 Monte Carlo simulations were applied to correct for multiple comparisons across surfaces using FreeSurfer. | 6min and 25s | 2,5s | 4×4×4 mm | N/I |
| Shu-xin Luan et al, 2018 | GRETNA & SPM | 6-mm | Age, gender, and gray matter volume | Threshold of p < 0.001, AlphaSim corrected | 6min | 2s | 3.75×3.75×3 mm | closed |
| Ting Ye et al, 2012 | MRIcro & SPM | 8-mm | N/I | Brain regions with significant changes of correlation coefficients were yielded at the voxel-level with a height threshold of p < 0.005 (uncorrected) | 5min | 2s | 3×3x3 mm | closed |
| Tobias Bracht et al, 2022 | FSL & CONN | 8-mm | Age, gender, mean FD, and mean DVARS | Voxel threshold of p < 0.001 and FWE cluster-level corrected p value <0.05 | 8min | 1s | 2.4×2.4×2.4 mm | closed |
| Wenbin Guo et al, 2015 | DPARSF & REST | 8-mm | Age, gender, education and head motion parameters | p>0.001 corrected for multiple comparisons using the Gaussian Random Field | 8min | 2s | 3×3×3 mm | closed |
| Xiaoping Wu et al, 2016 | AFNI & FSL | 6-mm | Motion parameters, white matter, and mean whole-brain signal. HDRS, HARS and ATQ scores | Corrected for multiple comparisons using cluster size Monte Carlo algorithms to obtain a significance level of po0.005. | 6 min and 15 s. | 2,5s | 4×4×4 mm | closed |
| Xiaolong Peng et al, 2018 | FSL & FreeSurfer | 6-mm | Head motion parameters, mean FD | Surface-based clusterwise correction for multiple comparisons was performed at the significance threshold of p < 0.01 | 6 min and15 s. | 2,5s | 4×4×4 mm | open |
| Xin-hua Yang et al, 2018 | DPARSF | 4-mm | Age, gender, IQ and frame wise displacement (FD) | Threshold of p < 0.05 AlphaSim corrected | 6min | 2s | 3.75×3.75×4.5mm | closed |
| Yanjun Meng et al, 2021 | FSL | 5-mm | Age, sex, baseline volume, cerebrospinal fluid ad white matter | Thresholded using cluster-forming correction determined by Z > 2.3 and a corrected cluster significance threshold of p < 0.05. | N/I | 5s | 3.75×3.75×4mm | N/I |

IQ, intelligence quotient; HDRS, Hamilton Depression Rating Scale; HARS, Hamilton Anxiety Rating Scale (HARS); ATQ, Automatic Thoughts Questionnaire scores; REST, resting-state fMRI data analysis toolkit - is a tool used to process data from resting-state functional magnetic resonance imaging; AFNI, a general statistical analysis for fMRI data; DPARSF, data processing assistant for resting-state fMRI; FSL is a comprehensive library of analysis tools for FMRI, MRI and diffusion brain imaging data; DPABI, a toolbox for Data Processing & Analysis of Brain Imaging; GRETNA, graph theoretical network analysis; MRIcro, is an application that allows you to view medical images; SPM, statistical parametric mapping; FDR, false discovery rate; BMI, body mass index; FD, framewise displacement; DVARS, is the root mean square of the temporal change of the fMRI voxel-wise signal at each time point (Yang et al, 2019); CONN, Cognitive and Affective Neuroscience Laboratory, Massachusetts Institute of Technology, Cambridge, MA; FWE, family wise error; GRE, Gaussian randon field correction; N/I, not informed.
