# Supplementary material for: Seed-based resting-state connectivity as a neurosignature in fibromyalgia and depression: a narrative systematic review
Source: Front Hum Neurosci. 2025 Apr 28;19:1548617. doi: 10.3389/fnhum.2025.1548617 (PMC12066659; doi:10.3389/fnhum.2025.1548617)
Supplement: Supplementary file 2 [file Table_2.docx]

Table B: the details regarding the rs-fMRI methodology in the Fibromyalgia studies (i.e. image acquisition parameters, quality control of head motion, multiple comparisons corrections, and covariates).

| **Study** | **Pre-processing and processing Software packages** | **Smoothing kernel size** | **Statistical covariates** | **Correction for multiple comparison** | **Scan duration/ volume no.** | **Repetition time** | **Voxel size** | **Eyes open/ closed** |
| --- | --- | --- | --- | --- | --- | --- | --- | --- |
| Andrea Truini et al, 2016 | FSL | 5 mm | Age | Images were thresholded using clusters determined by Z>3 (within group) and Z>2 (between group) and a corrected cluster significance threshold of p<0.05, including at least 20 contiguous voxels. | N/I | 3,32s | 1mm isometric | closed |
| Dajung Kim et al, 2021 | FSL, AFNI | 3.5-mm | Age | Cluster-extent threshold of p< 0.05 (FWE-corrected) | 6.8min | 3.5s | 1.9×1.9×3.5 mm | closed |
| Eric Ichesco et al, 2014 | FSL, SPM & CONN | 6-mm | Age | FWE cluster-level corrected p value <0.05 | 6-min | 2s | 3.3x2.4x4mm | open |
| Eric Ichesco et al, 2015 | SPM & CONN | 8-mm | Age &  scanning siteF | FWE cluster-level corrected p value <0.05 derived from a voxel-wise uncorrected threshold of p < 0.001. | 8 min | 2s | 3.12 x 3.12 x 3mm | open |
| Helene E. Veenstra et al, 2020 | SPM & CONN | 8-mm | HADS, PPT and pain intensity. | A significance threshold was applied with an initial cluster-defining voxel-based threshold of p < 0.001 uncorrected and a subsequent cluster-extent threshold of p < 0.05. | 10-min | 1.030s | 3x3x3mm | open |
| Ignacio Cifre et al, 2012 | FSL | 5-mm | N/I | N/I | 10min | 2,5s | 3×3×3mm | closed |
| Jesus Pujol et al, 2014 | SPM | 8-mm | N/I | Spatial extent thresholds by Monte Carlo  simulations and FWE rate correction of p < 0.05. | 6-min | 2s | 3.3x2.4x4mm | closed |
| Jian Kong et al, 2021 | CONN | 6 mm | Age & gender | FWE-corrected cluster threshold of p < .05. Monte Carlo simulation using 3dFWHMx and 3dClustSim was applied, and voxel-wise p<0.005 and p<0.05 at cluster level were corrected for the minimum voxel | 8 min 21s | 3s | 2.6×2.6×2.6mm | open |
| Jian Kong et al, 2019 | SPM & CONN | 8 mm | Age, gender & BDI-II | FWE-corrected cluster threshold of p < .05. Small volume correction to correct the p value | 8 min 21s | 3s | 2.6×2.6×2.6mm | open |
| Marie-Andree Coulombe et al, 2017 | FSL & fMRISTAT | 4-mm | N/I | FMRIB’s mixed effects thresholded at Z = 2.3 and a cluster-based p > 0.05. Bonferroni correction | 10.5 min | 3s | 3.13x3.13x3mm | closed |
| Marta Ceko et al, 2020 | FSL | 5 mm | N/I | Voxel-based threshold of z > 2.3 and cluster-corrected for spatialextent at p < 0.05 across the whole brain | 6-8 min | 2s | 3.5×3.5×3.5mm | open |
| Marta Ceko et al, 2013 | SPM & CONN | 8-mm | Age | Voxel wise threshold of p > 0.001 across the whole brain cluster-corrected for multiple comparisons at p > 0.05 | 8-min | 2,2s | 3.5×3.5×3.5mm | N/I |
| Nguyen Nhu et al, 2022 | CONN | 8-mm | Age and sex. | The voxel threshold was selected at a 1-sided P < 0.001, and the cluster threshold was set at P < 0.05 with family-wise error (FWE) correction. | N/I | 2.72s | 1x1x1mm | closed |
| Nicholas Fallon et al, 2016 | SPM & CONN | 8-mm | N/I | FDR-corrected cluster threshold of p < .05 | 20-min | 2s | 3×3×3.5mm | closed |
| Par Flodin et al, 2014 | FSL, SPM | 8-mm | Age, mean FD | The results are corrected at cluster level significance of FDR p < 0.05/159 = 0.00031, accounting for 159 t-tests using Bonferroni correction. Cluster defining voxel threshold was p < 0.001, uncorrected. | 7 min | 2.5s | 1x1x1mm | closed |
| Su Hyoun Park et al, 2022 | SPM & CONN | 4-mm | Age, pain duration | FDR corrected p-values p<0.05 | 12min | 2s | 2.9×2.9×2.9mm | N/I |

FSL is a comprehensive library of analysis tools for FMRI, MRI and diffusion brain imaging data; FMRIB stands for the Oxford Centre for Functional Magnetic Resonance Imaging of the Brain; CONN, Cognitive and Affective Neuroscience Laboratory, Massachusetts Institute of Technology, Cambridge, MA; SPM, statistical parametric mapping; FDR, false discovery rate; FEW, family wise error; fMRISTAT, a general statistical analysis for fMRI data; HADS, Hospital Anxiety and Depression Scale; HDRS, Hamilton depression rating scale; BDI-II, Beck Depression Inventory second edition; FD, framewise displacement; PPT, pressure pain thresholds; N/I, not informed
