# Supplementary material for: Seed-based resting-state connectivity as a neurosignature in fibromyalgia and depression: a narrative systematic review
Source: Front Hum Neurosci. 2025 Apr 28;19:1548617. doi: 10.3389/fnhum.2025.1548617 (PMC12066659; doi:10.3389/fnhum.2025.1548617)
Supplement: Supplementary file 3 [file Table_3.docx]

Table C: The details regarding the sample characteristics, clinical variables, and major findings of FC from MDD studies.

|  | **Sample size** | | **Mean Age ±SD**  **(Age range)** | |  |  | **Depression Symptoms** | | |  |  |  |  |  |
| --- | --- | --- | --- | --- | --- | --- | --- | --- | --- | --- | --- | --- | --- | --- |
| **Lead author and year** | **MDD (F)** | **HC (F)** | **MDD** | **HC** | **L-R** | **Dx tool** | **Depression Symptoms Mesuare** | **Mean (SD)** | **Mean condition duration (SD) (months)** | **Medication**  **status** | **% first episode patients** | **Seed Region** | **Major Findings (MDD vs HC):**  **↑ rs-FC** | **Major Findings (MDD vs HC):**  **↓ rs-FC** |
| Aixia Zhang et al, 2020 | 25 (13) | 26 (12) | 32.08 ±8.91 (18-50) | 32.19 ±8.64 | 0-25 | DSM-IV | HDRS-17 | 27.82 ±4.86 | N/I | Drug-naive MDD patients | First episode of MDD. | Bilateral AMY | ↑ rs-FC: IFG, right MFG, and the right MCC and paracingulate gyri | - |
| Antonie K Rubart et al, 2022 | 32 (22) | 40 (21) | 36.25 ±11.82 (18-65) | 40.2 ±13.56 | 3-29 | DSM-IV | HDRS-24 | 24.06 ±5.49 | >= 24 | Free of any medication for a minimum of two weeks before the study. | Patients with recurrent MDD. | PCC, ACC, precuneus, AMY, insula, MFG, THA, subcallosal anterior cingulate | Precuneus ↑ rs-FC: right SFG, right MFG, left MFG and IFG, left lateral occipital cortex | - |
| Chao Wang et al, 2018 | 23 (14) | 34 (19) | 30.48 ±7.13 (N/I) | 29.71 ±7.09 | 0-23 | DSM-IV | HDRS-24 | 34.3 ±7.58 | 43.04 ±58.18 | Free of any antidepressant.  Medication in the recurrent episode | 73,9% first episode of MDD and 26,1% patients with recurrent MDD. | Bilateral insula subregions | left dorsal agranular insula ↑ rs-FC: left IPL right ventral dysgranular and granular insula ↑ rs-FC: left THA and habehula | Left hypergranular insula ↓ rs-FC: sgACC |
| Daihui Peng et al, 2015 | 16 (9) | 16 (9) | 34.4 ±6.72 (25-50) | 33.75 ±6.36 | 0-16 | DSM-IV | HDRS-24 | 30.88 ±7.69 | 2 ±1 | Drug-naive MDD patients | First episode of MDD. | Bilateral precuneus | - | ↓ rs-FC: bilateral fusiform gyrus, right SMA, left precentral and postcentral gyrus |
| Jun Hu et al, 2021 | 114 (73) | 112 (72) | 39.07 ±12.8 (18-75) | 37.12 ±13.27 | N/I | DSM-IV | HDRS-17 | 20.72 ±4.3 | 50.1 ±61.74 | 56.14% Anti-depressant  43.85% Drug naive MDD patients | 83.3% first episode of MDD and 16.6% patients with recurrent MDD | Hippocampus subregions | - | Right anterior hippo ↓ rs-FC: right posterior insula, left postcentral gyrus, and right lateral orbito frontal cortex. Bilateral intermediated hippo ↓ rs-FC: left striatum (putamen and caudate), left IPL, left STL,left DLPFC, and lingual regions |
| Liu Kai et al, 2019 | 25 (19) | 35 (27) | 30 *  (19-64) (18-65) | 31  (19-61) | 0-25 | DSM-IV | HDRS-24 | 29  (17-44) | 2  (0.5-48) | Free of any medication for a minimum of 1 month before the study | 44% first episode of MDD and 56% patients with recurrent MDD | Bilateral striatum | ↑ rs-FC: mPFC and bilateral MTC and STC. | - |
| Ma Yue et al, 2024 | 34 (17) | 34 (16) | 49.52 ±12.61 (18-65) | 33.7 ±13.22 | 0-34 | CID-10 | HDRS-17 | 15.67 ±3.5 | N/I | Drug-naive MDD patients | N/I | Left insula | - | ↓ rs-FC:right MFG, right SFG, orbital part and right ACC |
| Qiang Wei et al, 2020 | 23 (16) | 20 (12) | 37.12 ±11.53 (18-65) | 38.5 ±10.32 | N/I | DSM-IV | HDRS-17 | 23.18 ±4.18 | N/I | 78.26% SSRI 60.8%Antipsychotics 39.1% SNRI  21.7% NaSSA 17.39% Anti convulsants  17.39% Antianxiety 17.39% Nonbenzo-diazepine hypnotic 4.37% NRI 4.37%.SARIs | N/I | left pulvinar THA | ↑ rs-FC: left precuneus | - |
| Qiaoying Zhang et al, 2022 | 36  (20) | 36  (21) | 34.32 ±5.45 (18-45) | 32.38 ±9.24 | 0 -36 | DSM-IV | HDRS-17 | 23.6 ±3.64 | N/I | Drug-naive MDD patients | First episode of MDD. | Bilateral sgACC | - | ↓ rs-FC: Hippo, PCC, AG, THA, striatum, insula, MTG, SFG, STG, and cerebellum |
| Shu-xin Luan et al, 2018 | 15  (6) | 15 (8) | 34.4 ±6.2 (18-55) | 33.5 ±6.8 | 0-15 | DSM-IV | HDRS-17 | 27.5 ±2.9 | 82.8 ±39.6 | Antidepressant with standard dosage and duration 100% | Patients with treatment resistant depression. | Bilateral habenular nucleus | Right habelunar nucleus ↑ rs-FC: medial SFG, ACC and medial orbitofrontal gyrus Left habenular nucleus ↑ rs-FC: ITG. | Right habelunar nucleus ↓ rs-FC: corpus callosum Left habelunar nucleus ↓ rs-FC: insula |
| Tobias Bracht et al, 2022 | 56 (26) | 22 (10) | 43.64 ±12 (18-65) | 42.91 ±13 | 6-47-3 | MINI DSM-IV | HDRS-21 | 21.69 ±5 | 12 ±11 | 38% Dual Antidepressants 35% Lithium,  23% Tricyclic antidepressants, 18% SSRI | Patients with recurrent MDD. | Bilateral VTA | ↑ rs-FC: right mPFC | - |
| Ting Ye et al, 2012 | 22 (14) | 30 (19) | 46.7 ±8.9 (18-59) | 45.9  ± 9 | 0-22 | DSM-IV | HDRS-17 | 18.5 ±6.3 | 8.6 ± 6.5 | Antidepressants (SSRIs), tricyclic antidepressants, and/or other hypnotics 22,72%. | First episode of MDD. | Right DLPFC | ↑ rs-FC: left dorsal anterior ACC, left PHG, THA and precentral gyrus | ↓ rs-FC: right PL |
| Wenbin Guo et al, 2015 | 44 (22) | 44 (24) | 27.52 ±8.57 (N/I) | 29.39 ±6.7 | 0-44 | DSM-IV | HDRS-17 | 25.18 ±5.22 | 19.61 ±36.5 | Drug-naive MDD patients | Patients with recurrent MDD. | Bilateral insula | - | Right insula ↓ rs-FC: left MFG, left STG, right putamen, and right mOccipital Gyr. Left insula  ↓ rs-FC: left STP and right mOccipital Gyr. |
| Xiaolong Peng et al, 2018 | 19 (10) | 19 (10) | 33.58 ±9.11 (18-45) | 33.89 ±8.88 | 0-19 | DSM-IV | HDRS-17 | 24.89 ±3.78 | N/I | Drug-naive MDD patients | First episode of MDD. | Insula subregions | Left ventral anterior insula ↑ rs-FC: inferior prefrontal gyrus, STS, AMY, Left dorsal anterior insula ↑ rs-FC: STS | Right posterior insula ↓ rs-FC: PPC posterior parietal cortex |
| Xiaoping Wu et al, 2016 | 19 (10) | 19 (10) | 34.32 ±5.4 (18-45) | 33.47 ±8.96 | 0-19 | DSM-IV | HDRS-17 | 24.89 ±3,78 | N/I | Drug-naive MDD patients | First episode of MDD. | Dorsal ACC | ↑ rs-FC: left precentral gyrus and AG | ↓ rs-FC: Bilateral MFG |
| Xin-hua Yang et al, 2018 | 40 (19) | 36 (19) | 28.75 ±6.67 (N/I) | 29.08 ±8.5 | N/I | DSM-IV | HDRS-24 | 28.73 ±4.88 | 6.16 ± 4.6 | Drug-naive MDD patients | First episode of MDD. | Bilateral ventral caudate nucleus and superior temporal gyrus | Ventral caudate  ↑ rs-FC: cuneus STG ↑ rs-FC: precuneus, AG and the cuneus | Ventral caudate  ↓ rs-FC: SFG, SPL and MTG |
| Yanjun Meng et al, 2021 | 30 (22) | 18 (12) | 28.83 ±10.15 (18-50) | 27.11 ±6.34 | 0-30 | DSM-IV | HDRS-24 | 16.43 ±6.03 | N/I | Not receive standardized drug treatment. | N/I | NAcc, hippocampus | - | NAcc ↓ rs-FC: PCC and precuneus |

DLPFC, dorsolateral prefrontal cortex; mPFC, medial prefrontal cortex; NAcc, nucleus accumbens; VTA, ventral tegmental area; MCC, middle cingulate cortex; PCC, posterior cingulate cortex; PHG, parahippocampal gyrus; ITG, inferior temporal gyrus; STS, superior temporal sulcus; AG, angular gyrus, ACC, anterior cingulate cortex; AMY, amygdala; IFG, inferior frontal gyrus; MTG, middle temporal gyrus; MFG, middle frontal gyrus; SFG, superior frontal gyrus; STP, superior temporal pole; STC, superior temporal cortex; STG, superior temporal gyrus; MTC, medial temporal cortex; STL, superior temporal lobe; Hippo, hippocampus; SMA, supplementary motor area; THA, thalamus; SPL, superior parietal lobule; IPL, inferior parietal lobe; Sg, subgenual; PL, parietal lobe; PPC, posterior parietal cortex; mOccipital Gyr, middle occipital gyrus; HC, healthy controls; MDD, Major depressive disorder; SNRI, serotonin-norepinephrine reuptake inhibitors; SSRI, selective serotonin reuptake inhibitors; NaSSA, noradrenergic and specific serotonergic antidepressant; NRI, noradrenaline reuptake inhibitor; SARI, serotonin antagonist and reuptake inhibitor; DSM, Diagnostic and Statistical Manual of Mental Disorders; HDRS, Hamilton Depression Rating Scale; N/I, not informed; SD, Standard deviation*.**Age expressed as median.
