# Supplementary material for: Seed-based resting-state connectivity as a neurosignature in fibromyalgia and depression: a narrative systematic review
Source: Front Hum Neurosci. 2025 Apr 28;19:1548617. doi: 10.3389/fnhum.2025.1548617 (PMC12066659; doi:10.3389/fnhum.2025.1548617)
Supplement: Supplementary file 4 [file Table_4.docx]

Table D: The details regarding the sample characteristics, clinical variables, and major findings of FC from Fibromyalgia studies.

|  | **Sample size** | | **Mean Age ±SD**  **(Age range)** | |  |  | **Fibromyalgia Symptom** | | | | |  |  |  |  |
| --- | --- | --- | --- | --- | --- | --- | --- | --- | --- | --- | --- | --- | --- | --- | --- |
| **Lead author and year** | **FM (F)** | **HC**  **(F)** | **FM** | **HC** | **L - R** | **Dx tool** | **FIQ Mean (SD)** | **Pain Intensity Mean (SD) NPS/ NRS/ VAS (cm)** | **Depression Symptom Mesuare** | **Mean (SD)** | **Mean condition duration (SD) (Years)** | **Medication status** | **Seed Region** | **Major Findings (FM vs HC):**  **↑ rs-FC** | **Major Findings (FM vs HC):**  **↓ rs-FC** |
| Andrea Truini et al, 2016 | 20 (19) | 15 (13) | N/I  (28-67) | N/I | N/I | ACR 2010 | N/I | N/I | ZSDS | N/I | N/I | No medication | Bilateral PAG | ↑ rs-FC: insula, ACC, and anterior pré-frontal cortex. | - |
| Dajung Kim et al, 2021 | 19 (19) | 21 (21) | 44.9 ±8.3 (30-60) | 45  ±8.4 | N/I | ACR, 1990 | N/I | VAS 52.8 ±20.3 | BDI | 19 ±6.8 | 2,96  ±0,25 | 74% Analgesics,  NSAIDs,muscle relaxants/  74% Anti-depressants; 37% Anti-convulsant | Posterior THA | ↑ rs-FC: Lef IPL Right IPL; Lef MTG and ITG |  |
| Eric Ichesco et al, 2014 | 18 (18) | 18 (18) | 35.8±12 (18-75) | 32.3  ±11.3 | 0-18 | ACR, 1990 | N/I | VAS 4.4  ±2.3 | N/I | - | 3.9  ±3.7 | N/I | Anterior, mid, and posterior Insular Cortex | Mid insula ↑ rs-FC: right MCC and PCC  Right posterior insula ↑rs-FC: left cingulate cortex Right anterior insula ↑rs-FC: left STG | Left anterior insula ↓rs-FC: bilateral MFG and ACC  Left posterior insula ↓rs-FC: right SFG |
| Eric Ichesco et al, 2015 | 12 (12) | 15 (15) | 38.5±12.1 (18-75) | 39.9  ±13 | 0-12 | ACR, 1990 | N/I | VAS 5.2 (2.0) | HADS depression | 6.9 ±3.9 | >= 1 | N/I | Lateral/ posterior THA and anterior  posterior insula | - | - |
| Helene E.-Veenstra et al, 2020 | 31 (31) | 28 (28) | 39.2±11.4  (22-56) | 42.64 ±10.24 | N/I | ACR, 1990 | 42.64 ±10.24 | NRS 5.68 ±1.83 | HADS-depression | 6 ±3.61 | N/I | NSAID, pain and sleep medication | PCC vmPFC, left IPS right IPS Left anterior insula Right anterior insula | ↑ rs-FC - Higher level of FC strength of right posterior IPS with right precentral gyrus. /↑ rs-FC: right IPS and the right anterior insula./ ↑ rs-FC: right posterior IPS seed with left insula and TL for FM>HC | - |
| Ignacio Cifre et al, 2012 | 9  (8) | 11  (9) | 52.3±8.9 (33-65) | 49  ±12.1 | 0-9 | ACR 2010 | N/I | N/I | BDI-II | 29.78 ±12.07 | 26.8  ±17.4 | 88.8% Anti-depressants 88.8% Anti-anxiety  44.4% NSAID Analgesic/ Muscle Relaxants. | ACC, PCC, AMY, CAU, PUT, GP, insula, M1 and SMAs, SI and SII, mPFC, PAG STS, and THA | ACC ↑rs-FC: the insula and BG/  SII ↑rs-FC: CAU/ M1 ↑rs-FC: SMA/ GP ↑rs-FC: AMY and mPFC ↑rs-FC: PCC | ACC ↓ rs-FC: AMY and PAG  THA ↓ rs-FC: insula and PAG/ Insula ↓rs-FC: PUT  PAG ↓rs-FC: CAU/ SII ↓rs-FC: motor cortex and PCC /PCC ↓rs-FC: STS |
| Jesus Pujol et al, 2014 | 40 (40) | 36 (36) | 46.4±7.5 (N/I) | 44  ±6 | 0-40 | ACR, 1990 | 66.2  ±14.2 | NRS 7  ±1,5 | HADS depression | 8.9 ±4.8 | 7.2  ±4.7 | 65% Anti-depressants 42.5% Anxiolytics  25% Gabapentin 20% NSAID 12.5% Opioids 5% Hypnotics 2.5% Paracetamol (Free from analgesic drugs 72h prior to fMRI) | POp/SII, and the auditory, visual,  SI, PAG, anterior, middle and posterior POp. | POp /SII ↑rs-FC: PCC, precuneus, ventral PUT, and ventral insula | PAG ↓rs-FC: anterior bilateral insula, left AMY, and right THA  POp /SII ↓rs-FC: auditory cortex, visual cortex, SI and posterior insula. |
| Jian Kong et al, 2021 | 20 (19) | 19 (19) | 51.6±11.6 (>=21) | 52.3  ±10.4 | N/I | ACR 2010 | 45.9  ±17,6 | N/I | BDI-II | 17.7 ±9.3 | N/I | N/I | Bilateral medial and lateral hypoth. | ↑ rs-FC medial hypoth: bilateral subcallosal cingulate cortex  ↑ rs-FC lateral hypoth right TP | ↓ rs-FC medial hypoth: bilateral cerebellum, AMY and right THA/ ↓ rs-FC lateral hypoth: right IOG /cerebellum |
| Jian Kong et al, 2019 | 21 (20) | 20 (19) | 53.1±11.58 (>=21) | 52.9  ±11.12 | N/I | ACR 2010 | 45.1  ±18.6 | N/I | BDI-II | 19.71 ±11.12 | N/I | N/I | Bilateral DLPFC | ↑ rs-FC: bilateral rostral anterior cingulate cortex and mPFC | - |
| Marie- Andree Coloumbe et al, 2017 | 23 (23) | 16 (16) | 50.6±8.1 (19–70) | 49.8  ±11.0 | N/I | ACR, 1990 | 60.3  ±15.8 | N/A | HADS depression | 7.3 ±3.4 | N/I | N/I | PAG | ↑ rs-FC: left lingual gyrus, PCC, cuneus, retrosplenial cortex and hippo. | ↓rs-FC: AG/lateral occipital cortex, PCC, PMC/SMA DLPFC) dmPFC and vlPFC. |
| Marta Ceko et al, 2013 | 28 (28) | 28 (28) | 48.7±7.8 (29-60) | 48.8  ±7.7 | 1 - 27 | ACR 2010 | N/I | VAS 2.6 ±2.7 Pressure sensitivity | HADS depression | 5.7  ± 4.3 | 11.5  ± 8.7 | 85.7% NSAID 46.4% Anti-depressants 25% Anti convulsants 17.8% Muscle Relaxants 7.14%Triptans 3.57% Cannabinoids | PCC, left anterior insula and NAcc | Younger FM patients NAcc ↑rs-FC: cluster located in DLPFC and PMC | Younger FM patients  Left anterior insula ↓rs-FC: dACC, TC, SI/M1 Older FM patients PCC ↓rs-FC: mPFC |
| Marta Ceko et al, 2020 | 27  (25) | 27  (25) | 42.3±13.1 (N/I) | 41.8  ±12.3 | N/I | N/I | N/I | 0 (0) | HADS depression | 4.76 ±3.02 | 10.6  ±7.72 | 25.9% NSAID 22.2% Anti-depressants  4.8% Muscle Relaxants 11.1% Anti-anxiety  3.7% Triptans | PCC, mPFC and PCC+ mPFC | - | ↓ rs-FC: left PUT |
| Marta Ceko et al, 2020 | 16 (16) | 16 (16) | 48.7±7.8 (N/I) | 48.8  ±7.7 | N/I | N/I | N/I | NRS 4.4  ±2.1 | HADS depression | 5.29 ±3.75 | 11.5  ±10.04 | 81.25% NSAID 31.25% Anti-depressants 18.75% Anti convulsants 6.25% Muscle Relaxants | PCC, mPFC and PCC+ mPFC | ↑ rs-FC: vermis cerebellum | - |
| Nguyen Nhu et al, 2022 | 26 (25) | 30 (28) | 49.6 ±11 (N/I) | 52.1 ±10.1 | N/I | ACR, 2016 | 53.1  ±16 | VAS 5.6  ±2.3 | BDI-II | 18.7 ±12.9 | N/I | Patients with FM have received stable medication dosages | VNM  VON | ↑ rs-FC - Right LG, Right OFusG, Small/not labeled | ↓rs-FC: Left FOrb , Left TP, Small/not labeled.  ↓rs-FC: Right FOrb, SubCalC Right NAcc, Small/not labeled |
| Nicholas Fallon et al, 2016 | 16 (16) | 15 (15) | 38.45±8.45 (N/I) | 39.4  ±8.65 | N/I | ACR, 1990 | 62.37 ±15.84 | N/I | BDI-II | 19.5 ±11.19 | 9.13  ± 6.80 | 68.75% doses of common medications with minimal central nervous efficacy. | PCC, precuneus, mPFG, bilateral IPL, right MTG and vACC | IPL ↑rs-FC: right hippocampal formation Left MFG ↑rs-FC: left PPC/ PCC ↑rs-FC: left anterior MCC. | PCC ↓rs-FC: right PHG, and right ITG. |
| Par Flodin et al, 2014 | 16 (16) | 22 (22) | 48.3 *  (25-64)  (20-65) | 45.7 (20-63) | N/I | ACR, 1990 | 61.2 ±13.3 | N/I | N/I | N/I | 7.6  ±3.8 | 68,7% Anti depressants; 6% Anti convulsant | Insula, SMgyr, MCC, THA | ↑ rs-FC: SMgyr and Cerebellum | ↓ rs-FC: Insula - SI/M1  ↓ rs-FC: SMgyr -SI/M1 and inferior PFC  ↓ rs-FC: Mid cingulate - Occipital cortex, dmPFC, PCC ↓ rs-FC: THA and PreMC |
| Su Hyoun Park et al, 2022 | 32 (32) | 37 (37) | 41.9 ±12.7 (N/I) | 48.4 ±10.6 | 2-30 | ACR, 2010 | N/I | BPI 5.2  ±2 | BDI | 16.3 ±8.8 | 9.08  ±7.35 | 33% mood-altering or pain medications. 25% NSAIDs, 21% GABA, 21% SNRIs, 12% TCAs, 12% SSRIs, 6% LDN,  6% anti-convulsant, 12% muscle relaxants  9% other anxiolytics, 3% medical cannabis  3% triptans, 3%benzo diazepine  3% use topical lidocaine patches. | right NAcc | - | ↓rs-FC: bilateral mPFC, left PUT, left THA, and left ventral pallidum. |

FM, Fibromyalgia; ACR, American College of Rheumatology; FIQ, Fibromyalgia Impact Questionnaire; HC, healthy controls; VAS, visual analog scale; NRS, Numeric Rating Scale; NPS, Numeric Pain Scale; ZSDS, Zung Self-Rating Depression Scale; HADS, Hospital Anxiety and Depression Scale; BDI-II, Beck Depression Inventory second edition; BPI, Brief Pain Inventory; NSAIDs, nonsteroidal anti-inflammatory drugs; SNRI, serotonin-norepinephrine reuptake inhibitors; SSRI, selective serotonin reuptake inhibitors; NaSSA, noradrenergic and specific serotonergic antidepressant; NRI, noradrenaline reuptake inhibitor; SARI, serotonin antagonist and reuptake inhibitor; GABA, gamma-aminobutyric acid analogs; TCA, tricyclic antidepressants; LDN, low-dose naltrexone; DLPFC, dorsolateral prefrontal cortex; mPFC, medial prefrontal cortex; mPFG, medial prefrontal gyrus; NAcc, nucleus accumbens; MCC, middle cingulate cortex; PCC, posterior cingulate cortex; PHG, parahippocampal Gyrus; ITG, inferior temporal gyrus; STS, superior temporal sulcus, M1, primary motor cortex; SI/SII; primary and secondary somatosensory cortex; ACC, anterior cingulate cortex; dACC, dorsal anterior cingulate cortex; PAG, periaqueductal gray; AMY, amygdala; MFG, middle frontal gyrus; SFG, superior frontal gyrus; STG, superior temporal gyrus; TL, temporal lobe; TC, temporal cortex; TP, temporal pole; Hypoth, hypothalamus; SMA, supplementary motor area; THA, thalamus; dmPFC, dorsomedial prefrontal cortex; vmPFC, ventromedial prefrontal cortex; vlPFC, ventrolateral prefrontal cortex; IPL, inferior parietal lobe; GP, globus pallidum; CAU, caudate; PUT, putamen; IOG, inferior occipital gyrus; POp/SII: Parietal operculum/SII; VNM, visual medial network; VON, visual occipital network; SMgyr, supramarginal gyrus; MTG, middle temporal gyrus; AG, angular gyrus; IPS, intraparietal sulcus; PreMC, premotor cortex; PFC, prefrontal cortex; BG, basal ganglia; PPC, posterior parietal cortex; PMC, posteromedial cortex; LG, lingual gyrus; OFusG, occipital fusiform gyrus; FOrb, frontal orbital cortex; Hippo, hippocampus; N/I, not informed; SD, Standard deviation.*Age expressed as median.
